# Flagella-Associated WDR-Containing Protein CrFAP89 Regulates Growth and Lipid Accumulation in *Chlamydomonas reinhardtii*

**DOI:** 10.3389/fpls.2018.00691

**Published:** 2018-05-29

**Authors:** Qiulan Luo, Wenwen Song, Yajun Li, Chaogang Wang, Zhangli Hu

**Affiliations:** ^1^Guangdong Technology Research Center for Marine Algal Bioengineering, Guangdong Key Laboratory of Plant Epigenetic, Shenzhen Key Laboratory of Marine Bioresource & Eco-environmental Sciences, College of Life Sciences and Oceanography, Shenzhen University, Shenzhen, China; ^2^Key Laboratory of Optoelectronic Devices and Systems of Ministry of Education and Guangdong Province, College of Optoelectronic Engineering, Shenzhen University, Shenzhen, China; ^3^Key Laboratory of Tropical Crop Biotechnology, Ministry of Agriculture, Institute of Tropical Bioscience and Biotechnology, Chinese Academy of Tropical Agricultural Sciences, Haikou, China; ^4^College of Plant Health and Medicine, Qingdao Agricultural University, Qingdao, China; ^5^Shenzhen Engineering Laboratory for Marine Algal Biotechnology, Longhua Innovation Institute for Biotechnology, Shenzhen University, Shenzhen, China

**Keywords:** E3 ubiquitin ligase, WDR proteins, lipid metabolism, triacylglycerol, RNA interference silencing

## Abstract

WD40-repeat (WDR) domain-containing proteins are subunits of multi-protein E3 ligase complexes regulating various cellular and developmental activities in eukaryotes. *Chlamydomonas reinhardtii* serves as a model organism to study lipid metabolism in microalgae. Under nutrition deficient conditions, *C. reinhardtii* accumulates lipids for survival. The proteins in *C. reinhardtii* flagella have diverse functions, such as controlling the motility and cell cycle, and environment sensing. Here, we characterized the function of CrFAP89, a flagella-associated WDR-containing protein, which was identified from *C. reinhardtii* nitrogen deficiency transcriptome analysis. Quantitative real time-PCR showed that the transcription levels of *CrFAP89* were significantly enhanced upon nutrient deprivation, including nitrogen, sulfur, or iron starvation, which is considered an effective condition to promote triacylglycerol (TAG) accumulation in microalgae. Under sulfur starvation, the expression of *CrFAP89* was 32.2-fold higher than the control. Furthermore, two lines of RNAi mutants of *CrFAP89* were generated by transformation, with gene silencing of 24.9 and 16.4%, respectively. Inhibiting the expression of the *CrFAP89* gene drastically increased cell density by 112–125% and resulted in larger cells, that more tolerant to nutrition starvation. However, the content of neutral lipids declined by 12.8–19.6%. The fatty acid content in the transgenic algae decreased by 12.4 and 13.3%, mostly decreasing the content of C16:0, C16:4, C18, and C20:1 fatty acids, while the C16:1 fatty acid in the *CrFAP89* RNAi lines increased by 238.5 to 318.5%. Suppressed expression of TAG biosynthesis-related genes, such as CrDGAT1 and CrDGTTs, were detected in *CrFAP89* gene silencing cells, with a reduction of 16–78%. Overall our results suggest that down-regulating of the expression of *CrFAP89* in *C. reinhardtii*, resulting in an increase of cell growth and a decrease of fatty acid synthesis with the most significant decrease occurring in C16:0, C16:4, C18, and C20:1 fatty acid. *CrFAP89* might be a regulator for lipid accumulation in *C. reinhardtii*.

## Introduction

WD40-repeat (WDR) proteins comprise a diverse superfamily of regulatory proteins characterized by the presence of several WD motifs (also known as the Trp-Asp or WD40 motifs). Numerous WDR proteins have been found to play key roles in disparate mechanisms, such as signal transduction, cytoskeletal dynamics, protein trafficking, nuclear export, and RNA processing, and are especially prevalent in chromatin modification and transcriptional mechanisms (van Nocker and Ludwig, 2003). WD40 domains are considered as the key motifs of WDR proteins, which mediate diverse protein–protein interactions, and are involved in scaffolding as well as cooperative assembly and regulation of dynamic multi-subunit complexes (Stirnimann et al., 2010). WDR motifs constitute a series of sequences repeats of 44–60 residues with characteristic sequence and structural features, and are usually arranged in a β-propeller fold of six to eight “blades” that form a rigid interaction scaffold (Li and Roberts, 2001). The functions of several WDR proteins in eukaryotes have been well characterized, including RNA processing, organ differentiation, and abiotic stress responses (Jesús et al., 2005; Kong et al., 2015; Chen et al., 2016). Some WDR proteins are also known to regulate metabolic pathways in cells, for example, F-box and WD Repeat Domain-Containing 7 (FBW7) protein negatively regulates glucose metabolism in pancreatic cancer (Ji et al., 2016). The diverse functions of WDR proteins have also been characterized in plants, including abiotic stress responses, cell cycle control, signal transduction, and flowering (Zhao et al., 2007; Chuang et al., 2015; Gourlay et al., 2015); however, they are not well characterized in microalgae.

Microalgae have increasingly drawn the attention of academic and industrial researchers for using as a feedstock to produce biodiesel, owing to their significant advantages over other biofuels, including a high photosynthetic efficiency, fixation of CO_2_, less environment pollution and lack of competition with food production and agricultural resources (Larkum et al., 2012). However, the production of biofuel from microalgae is limited by its high cost. The main problem is the contrast between biomass productivity and lipid accumulation. How to resolve this contradiction has become an intractable problem in the production of biodiesel from microalgae. Some genetic or metabolic biotechnologies have been previously applied in microalgae breeding in an attempt to obtain an ideal strain to satisfy production requirements (Larkum et al., 2012). Microalgae biosynthesis can produce large amounts of lipids, predominantly triacylglycerols (TAGs), under stressful conditions, including starvation, salinity, heat, and et al, at the expense of cellular growth and biomass (Sun et al., 2014). Nitrogen (N) and sulfur (S) are essential macro-elements for microalgae culture. The deficiency of nitrogen or sulfur could markedly encourage lipids accumulations, especially TAGs, in microalgae cells (Cakmak et al., 2012). To reveal the mechanisms of lipids biosynthesis, some transcriptomics-, metabolomics-, and proteomics-based studies have been performed with *C. reinhardtii* under nitrogen- or sulfur-deficient conditions (Zhang et al., 2004; Park et al., 2015; Yang et al., 2015). *C. reinhardtii* has complex regulatory networks that respond to N deprivation, and N-sensing mechanisms play important roles in modulating the cell physiology in the levels of numerous transcripts, proteins, and metabolites (Park et al., 2015). During sulfur deprivation, photosynthetic apparatus in *C. reinhardtii* are restructured and its metabolic processes change in order to accumulate starch or lipids (Zhang et al., 2004).

The unicellular green alga, *C. reinhardtii*, is considered an ideal reference microorganism that provides valuable insight into the production of secondary metabolites owing to its sequenced genome and easy genetic transformation (Yamano et al., 2013). *C. reinhardtii* is a biflagellate alga; its flagella play important roles in controlling cell motility, sensing environmental cues, and mediating ciliary signal transduction (Long et al., 2015). Hundreds of proteins have been identified in *C. reinhardtii* flagellar, including a variety of E3 ubiquitin ligases, such as WDR-containing proteins, RING proteins, and cullins (Pazour et al., 2005). Posttranslational protein modifications, especially ubiquitination, are critical processes in cellular regulation. E3 ubiquitin ligase complexes (E3s) are responsible for substrate recognition in ubiquitination (Chen and Hellmann, 2013). WDR proteins are often conserved as key subunits in E3s, such as DDB1 (DNA damage-binding protein 1)-binding/WD40 repeat-containing (DWD) (Beris et al., 2016), which serve as adaptors interacting with the substrates. The function of WDR proteins in the flagella or ciliary still not known. Previous studies have shown that WDR proteins are specifically required to fold key components that are necessary to build motile ciliary axonemes (Patel-King and King, 2016). However, there are very few reports about WDR proteins in flagella helping to regulate cell growth and lipid metabolism.

Here, we demonstrate that a flagella-associated protein 89 (CrFAP89), whose expression was found to be enhanced in the nitrogen deficiency transcriptome, encodes an evolutionarily conserved WDR domain-containing protein, and is involved in regulating cell growth and lipid biosynthesis in *C. reinhardtii*. In eukaryotic cells, WDR proteins containing 4–16 tandem WD40 repeats are usually identified as E3s, which act as regulators and signaling hubs in many cellular and developmental processes (Stirnimann et al., 2010). Moreover, earlier studies have shown that WDR proteins also regulate the lipid metabolism via ubiquitin-mediated degradation of adipose (ADP), an important protein that regulates fat storage in *Drosophila* (Hader et al., 2003). However, there are very few studies on the role of WDR proteins in regulation of lipid biosynthesis in algae. Our study reveals that CrFAP89, an evolutionarily conserved WDR domain protein, is up-regulated under starvation conditions, with a deficiency of either nitrogen, sulfur, or iron. The RNAi silencing of *CrFAP89* promotes cell growth and nutrition starvation-tolerance but causes a reduction in lipids in *C. reinhardtii*. Previous studies have revealed that the biosynthesis of TAG in algae occurs mainly through acyl-CoA-dependent Kennedy pathway and acyl-CoA-independent pathway. Diacylglycerol acyl transferases (DGAT) that catalyzes the acylation of a diacylglycerol (DAG) with an acyl-CoA forming TAG were key enzymes in the Kennedy pathway (La Russa et al., 2012). Moreover, phospholipid: DAG acyltransferases (PDATs), which transfer a fatty acyl moiety from a phospholipid (PL) to DAG to form TAG was the limiting enzyme in another pathway (Driver et al., 2017). In this study, the expression of *DGAT* and *PDAT* genes in *CrFAP89* RNAi silencing lines have been detected. Finally, FAME profiling of *CrFAP89* RNAi silencing lines showed a diminished accumulation of fatty acids. On the basis of these results, we propose that CrFAP89 is potentially involved in cell growth and lipid accumulation.

## Materials and Methods

### Bioinformatic Sequence Analysis of CrFAP89

The sequence information of CrFAP89 (Cre01.g039500) was obtained from Phytozome version 12.0^1^ using version 5.5 of *C. reinhardtii* genome annotations. For identification of the putative domains in CrFAP89, the SMART program^2^ was used. Sequences of homologous proteins were retrieved from UniProtKB^3^ by BLASTP searching. Phylogenetic trees were generated by Mega7.0 (Kumar et al., 2016) using a ClustalW2 alignment and Neighbor-Joining (NJ) method. For bootstrapping, 1000 replicates were performed to establish the reliability of the NJ tree. The protein properties of CrFAP89, including molecular weight, isoelectric point, and hydrophobic properties, were analyzed by ExPASy^4^. Comparative 3D protein predictions were performed with the Phyre^2^; software^5^, and human apoptotic protease-activating factor 1 (APAF1, PDB ID: c5juyB, chain B, resolution of 4.1 Å) was selected as the model template (Cheng et al., 2016).

### *C. reinhardtii* Strain and Growth Conditions

*Chlamydomonas reinhardtii* cc425 (cell wall-deficient strain) was purchased from the *Chlamydomonas* Resource Center. Tris-acetate-phosphate (TAP) media was applied during cell transformation. Before starting cultivation in nutrient-deficient media, cells were cultured in Sueoka’s high-salt medium (HSM) and exposed to continuous illumination at an intensity of 25 μmol photons⋅m^-2^⋅s^-1^ in an orbital shaker at 24°C. Cells in the logarithmic phase were washed twice with sterilized water by centrifugation at 3000 × *g* for 5 min. Washed cells were cultured in either low-nitrogen (-N), low-sulfur (-S), or low-iron (-Fe) conditions. The components of the media are listed in the Supplementary Table S1.

### Transcription Analysis of *CrFAP89*

Total RNA was extracted from replicated samples of day 2, day 4, day 6, and day 8 cultures of -N, -S, or -Fe treated cells using TRIzol reagent (Invitrogen, Carlsbad, CA, United States). After reverse transcription, real-time PCR was carried out using a SYBR Premix Ex Taq Kit (TaKaRa Bio, Otsu, Japan) using Agilent StrataGene Mx3005P, with primer sets specific for *CrFAP89*. *18S rRNA* (GenBank No: MF101220.1) was used as an internal standard. All PCR primers are listed in Supplementary Table S2. The relative abundance of mRNA level was determined using the 2^-ΔΔCT^ method (Livak and Schmittgen, 2001). Fold change and standard error were calculated by averaging three replicates of log-transformed data. For expression analysis in *CrFAP89*i lines, genes encoding key enzymes in TAG biosynthesis were selected, including *CrDGAT1, CrDGTTs*, and *CrPEPC1*.

### Generation of *CrFAP89* RNAi Transgenic Strains

The vector for the RNAi-mediated silencing of the *CrFAP89* gene was constructed as described previously (Luo et al., 2015). The region between 3379th and 3666th nucleotide (including WD40 domain coding fragment) of *CrFAP89* full length cDNA was amplified by RT-PCR using primer pairs BamHI*CrFAP89*-Fi/HindIII *CrFAP89*-Ri (sense) and PstI *CrFAP89*-Fi/XbaI *CrFAP89*-Ri (antisense). After double digestion, the resulting fragments were cloned into the respective cloning sites of the pMD18T-18S flanking the 282 bp DNA spacer. The cassette was inserted into the EcoRI sites of Maa7/X IR by blunt end ligation. *C. reinhardtii* CC425 was subsequently transformed with the resulting RNAi vector p*Maa7*IR/*CrFAP89* by the glass bead method (Kindle, 1990). Transgenic cells were plated on selective media containing 1.5 mM L-tryptophan, 5 μg/mL paromomycin, and 5 μM 5-FI. CrFAP89 RNAi strains were picked out from the resistant colonies by quantitative RT-PCR.

### The Growth and Neutral Lipid Accumulation Kinetics Analysis

*CrFAP89* RNAi transgenic strains and negative controls (p*Maa7*IR/X transgenic strains) were grown in HSM for 9 days, and the cells densities were measured using a Coulter^TM^ Multisizer 4 (Beckman Coulter, Brea, CA, United States) in every 2 days. The examination of neutral lipid content (NL) was performed according to Chen et al. (2009). Cells were stained by 0.5 μg/mL Nile red dye in 200 μL solution containing 25% (v/v) DMSO for 10 min, and then fluorescence intensity (FI) was detected by a Glomax-Multi Detection System (Promega, Madison, WI, United States), with excitation and emission wavelengths of 530 and 575 nm, respectively. Triolein (Sigma, St. Louis, MO, United States) was used to make a standard curve. The NL (mg/mL) was calculated using the formula: NL = (0.0004 × FI – 0.0038) × 0.05/mL. In order to visualize the lipid accumulation in *CrFAP89*i strains, cells were cultured in HSM for 10 days, and then stained with Nile red and photographed using a Nikon 80i fluorescence microscope. The Nile red signals were captured at an excitation wavelength of 480 nm, and the emission was collected between 560 and 600 nm.

### FAME (Fatty Acid Methyl Ester) Profiling

Fatty acids (FA) were extracted from 5 to 10 mg dry cells (dried in a vacuum freezing machine) grown in HSM for 10 days. First, the dry biomass was incubated with 1 mL of 2 M sodium hydroxide-methanol solution for 1 h in a shaker at a speed of 100 × *g*, and then saponified at 75°C for 15 min. One microgram of methyl non-adecanoate (C15:0) was added as an internal standard. After saponification, the fatty acid was methyl-esterified with 1 mL 4 M hydrochloric acid-methanol incubated at 75°C for 15 min. Upon cooling, hexane (1 mL) was added into the vial after methylation to extract the methyl esters. After drying by nitrogen blowing, the samples were dissolved in 500 μL of CH_2_Cl_2_ and then analyzed by GC/MASS (Agilent 6890N, Tokyo, Japan).

### Statistical Analysis

One-way analysis of variance (ANOVA), followed by Duncan’s post-test was performed using the SSPS11.5 program in order to examine the significance in the differences between the means. Significant differences between the controls and other samples are indicated as follows: the different letters indicate that they were significantly different at *P* ≤ 0.05, ^∗∗^*P* ≤ 0.01 according to one-way ANOVA.

## Results

### *CrFAP89* Encodes a Highly Conserved WD40-Repeat-Containing Protein

**FIGURE 1:**
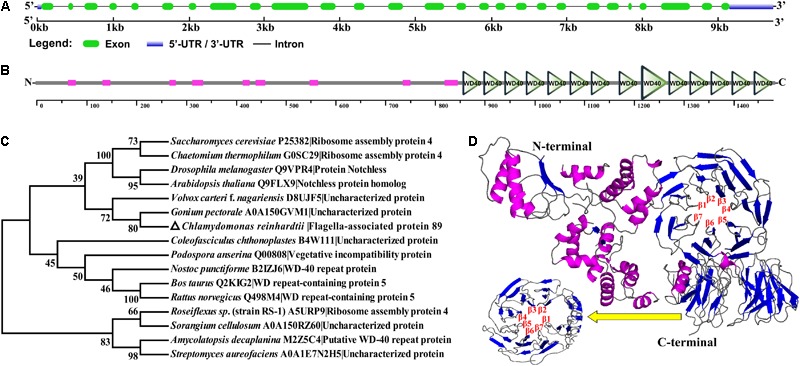
CrFAP89 was a novel WD40 repeat-containing protein. **(A)** Gene structure of the *CrFAP89*. The blue rectangle indicates the 5′- or 3′-UTR regions, the green boxes show exons, and the lines show the introns. **(B)** The WDR domain is a conserved sequence at the C-terminus of CrFAP89 shown as triangles, and magenta boxes show unnamed domains with low complexity. **(C)** Neighbor joining-phylogenetic unrooted tree of CrFAP89 constructed by MEGA7. **(D)** Predicted 3D protein structure of CrFAP89 using Phyre^2^ software. The magenta and blue lines show the α-helix and β-sheet, respectively, β1 to β7 denote the interaction hubs composed of the seven β-sheets. The yellow arrow points to one view of the second interaction hub.

The *CrFAP89* sequence, obtained from *C. reinhardtii* V5.5 in Phytozome, is located at chromosome_1:5555423–5565163 and contains 27 exons and 26 introns (**Figure 1A**). The coding region of CrFAP89 is 4,446 bp long and encodes a protein containing fourteen WD40 repeats in the C-terminal, which comprised almost half of it (**Figure 1B**). The theoretical pI and molecular weight of CrFAP89 are 6.75 and 157 kDa, respectively. Twelve potential transmembrane segments were predicted in CrFAP89 by the ‘DAS’ server, without any SignalP. Conserved amino acid sequences for CrFAP89 orthologs from various species were cataloged and used for BLAST in NCBI. The phylogenetic relationship of CrFAP89 with orthologs from various species was analyzed using the MEGA7 software by the neighbor-joining method. The CrFAP89 protein is evolutionarily conserved in all organisms from yeast to *Bos taurus*, and has novel WDR domains, including ribosome assembly protein 4 and WD-40 repeat-containing proteins. As shown in **Figure 1C**, CrFAP89 orthologs from 16 species were clustered into four groups. CrFAP89 from *C. reinhardtii* was closely aligned with orthologs from the green algae *Gonium pectoral* and *Volvox carteri* f. *nagariensis*. In 3D structure prediction, 76% of CrFAP89 sequence was modeled with 100.0% confidence by the template, with an identity of 21%. The fourteen WD40 domains at the C-terminal of CrFAP89 formed two conserved β-sheet structures (**Figure 1D**), denoting two central interaction hubs.

### Expression of *CrFAP89* Was Up-Regulated Under Nutrition Deficient Conditions

**FIGURE 2:**
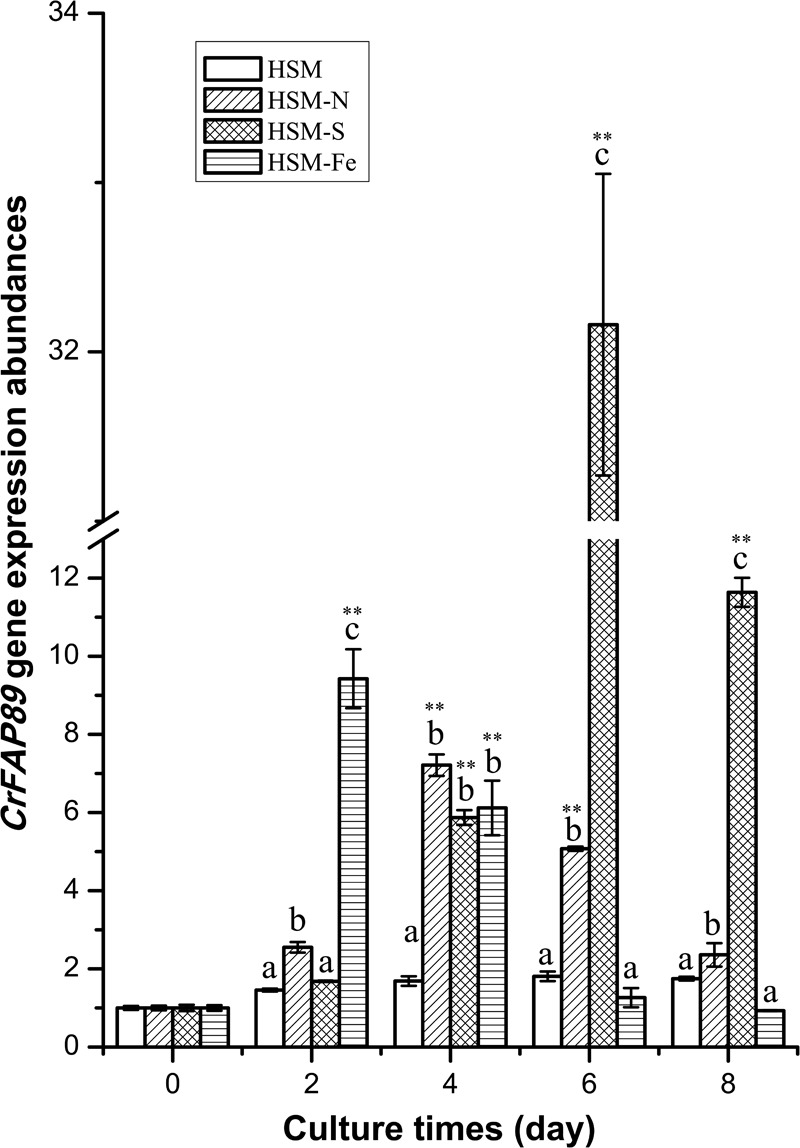
The expression patterns of *CrFAP89* under deprivation of nitrogen, sulfur, and iron. The different letters of each column indicates that they were significantly different at *P* ≤ 0.05, ^∗∗^*P* ≤ 0.01 according to one-way ANOVA.

To understand the physiological function of CrFAP89, its expression profiles under conditions of nutrition element deficiency, including nitrogen (N), sulfur (S), and iron (Fe), were analyzed by real-time quantitative PCR. As showed in **Figure 2**, *CrFAP89* was expressed steadily in algal cells grown under normal conditions. When algal cells were cultured in N, S, or Fe deficient media for 2 days, the transcript levels of *CrFAP89* were drastically up-regulated. In the absence of nitrogen, *CrFAP89* was up-regulated and reached its peak (7.2-fold) after 4 days of treatment, after which the expression level gradually declined. The deficiency of sulfur also strongly encouraged the transcription of *CrFAP89*, especially after treatment for 6 days with the highest expression level of 32.2-fold. When the cells were subjected to iron deficiency, the transcription levels of *CrFAP89* changed drastically, reaching its highest level after 2 days of treatment, and after which they decreased significantly. Thus, the expression profile of *CrFAP89* suggests that *CrFAP89* plays a key role in the nutrition deficiency response.

### Silencing of *CrFAP89* Encourages Cells Growth

**FIGURE 3:**
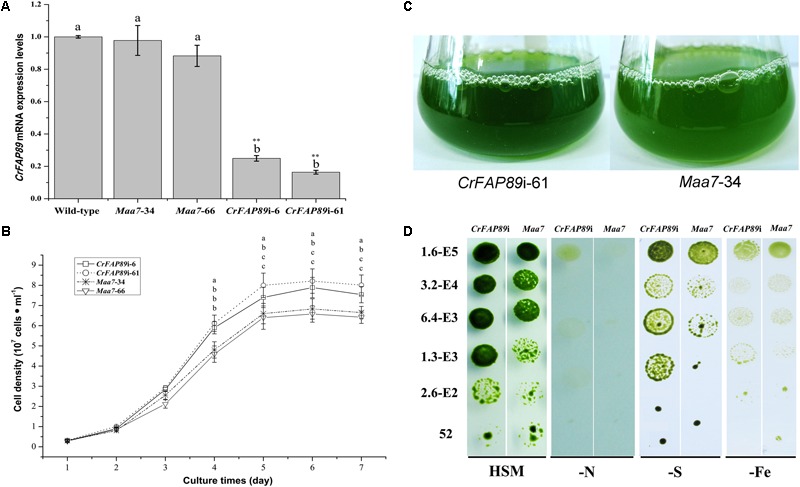
The growth of *CrFAP89*-RNAi algal lines. **(A)** RNA-mediated interference of *CrFAP89* in *CrFAP89*i-6, -61 compared with the control transformed with an empty vector (*Maa7*-34, 66) and wild-type (cc425), confirmed using real-time PCR. The different letters of each column indicate that they were significantly different at *P* ≤ 0.05, ^∗∗^*P* ≤ 0.01. **(B)** The growth curves and **(C)** liquid cultures of the *CrFAP89*-RNAi cell lines and the control transformed with an empty vector grown in HSM medium. The different letters of each lines indicate they were significantly different at *P* ≤ 0.05. **(D)** Growth of CrFAP89-RNAi and control lines under nutrition starvation conditions. Growth in HSM served as a control, while HSM with nutrition element deficiency, including low sulfur (–S), nitrogen (–N), and iron (–Fe), were used to induce stress. The numbers listed along the left indicate the initial inoculum cell density.

To illustrate the functional role of CrFAP89 protein in *C. reinhardtii*, a 288 bp-fragment encoding partial WDR domain was sub-cloned and then used to generate *CrFAP89* RNAi construct p*Maa7*IR/*CrFAP89*IR. Two *CrFAP89*-RNAi lines with lowest *CrFAP89* expression levels were picked out from over 100 paromomycin and 5-FI resistant transformants. The qRT-PCR analysis showed that the expression of *CrFAP89* in these RNAi-algal lines was significantly suppressed by 24.9 and 16.4% compared to the wild-type (**Figure 3A**), while its expression in transgenic lines transformed with empty vector showed only slight decline. These two RNAi lines were denoted as *CrFAP89*i-6 and *CrFAP89*i-61 and were used for further experiments.

To investigate the functional role of CrFAP89 in regulating cell growth, the cell densities of transgenic lines were examined using cell count technique. When the two *CrFAP89* RNAi-expressing algal lines were cultured in HSM, they showed an increase in cell number by about 112∼125% compared with the lines harboring empty vector; however, their growth rates were similar (**Figure 3B**). The differences in cells number between the *CrFAP89* RNAi-expressing algal lines and negative control appeared after 4 days of growth in HSM. All the algal lines reached their growth peaks at 5 days, when the population of *CrFAP89*i-61 has the most cells. After culturing for 7 days, the liquid of *CrFAP89*i-61 exhibited greener and thicker than *Maa7*-34 control line (**Figure 3C**), also showing the more biomass of *CrFAP89*i-61.

*CrFAP89*-interference transgenic cells were grown on HSM medium and other nitrogen-, sulfur-, or iron-deficient media for 1 week. The *CrFAP89*-RNAi transgenic lines were found to grow more quickly and efficiently than the control. As shown in **Figure 3D**, *CrFAP89*-interference lines show better growth under nutrition starvation conditions compared with the control lines. These results demonstrate that *CrFAP89* plays an important role in the algae’s response to nutrition starvation.

### Diminished Lipid Accumulation in *CrFAP89*-Silenced Algal Cells

**FIGURE 4:**
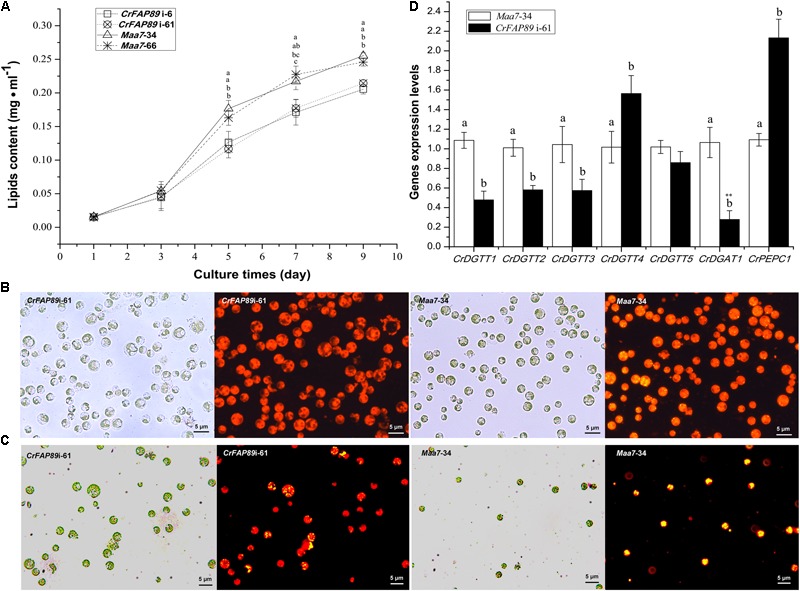
Lipid accumulation in *CrFAP89*-RNAi lines and control. **(A)** Lipid content in *CrFAP89*-RNAi lines and the control transformed with an empty vector grown in HSM medium. The different letters of each line indicate that they were significantly different at *P* ≤ 0.05. **(B)** Fluorescence microscopy images of *CrFAP89*-RNAi cells and control cells grown for 9 days in HSM and **(C)** after N-deprivation for 4 days to visualize TAG by Nile Red staining. Red indicates chlorophyll autofluorescence, and yellow indicates the lipid bodies. **(D)** Expression analysis of genes encoding key enzymes in lipid biosynthesis in *CrFAP89*-RNAi lines and control. The different letters of each column indicates they were significantly different at *P* ≤ 0.05, ^∗∗^*P* ≤ 0.01 according to one-way ANOVA.

*CrFAP89* transcripts were up-regulated by nitrogen deprivation, a condition known to result in the accumulation of lipids. In order to investigate whether CrFAP89 participates in lipid biosynthesis, we detected the lipid content in *CrFAP89*-RNAi algal lines using a fluorescence method. As shown in **Figure 4A**, when the expression of *CrFAP89* was suppressed in algal cells, the lipid content significantly decreased after 5 days of growth. The lipid content in both Cr*FAP89*i-6 and Cr*FAP89*i-61 lines was reduced by 21.2 and 22.1% after 7 days compared to the control lines transformed with an empty vector. The fluorescence microscopy images of the cells after Nile red staining visually demonstrated that there was a reduced number of lipid bodies in the transgenic *CrFAP89*-silenced lines. As shown in **Figure 4B**, there were very few lipid bodies in the *CrFAP89*i-61 line compared to control. Another interesting observation was that the cells of the *CrFAP89*i-61 seemed larger than the control. Furthermore, these cells line were stained with Nile red after 4 days nitrogen deprivation and photographed with fluorescence microscopy. The cells of the control lines *Maa7*-34 were almost filled with lipids bodies, whereas *CrFAP89*i-61 algal cells had less lipids bodies, meaning that lipid biosynthesis was discouraged when *CrFAP89* gene was suppressed (**Figure 4C**).

*DGAT* and phosphoenolpyruvate carboxylase (*PEPC*) gene families have been reported to play key roles in lipid biosynthesis (Kao and Ng, 2017). As such, we analyzed the expression patterns of these genes in the *CrFAP89*i-61 line using qRT-PCR assay. As shown in **Figure 4D**, when *CrFAP89* is silenced, the expression of *DGATs* declines by 16–78%, except for the *CrDGTT4* gene, which increases slightly. Also, the transcriptional level of *CrPEPC1* in the *CrFAP89*i-61 line was drastically up-regulated, about 2.1-fold compared to the negative control. These results suggest that the genes involved in lipid biosynthesis are likely regulated by CrFAP89 in algal cells.

### Silencing of *CrFAP89* Changes Fatty Acid Composition

After 9 days of growth in HSM medium, fatty acids were measured by GC/MS following extractive methylation to fatty acid methyl esters (FAMEs, **Table 1**). The main components of the fatty acids included C16:0, C16:1, C16:2, C16:4, C18:0, C18:1t, C18:2t, C18:3(5, 9, 12), and C18:3n3. Total fatty acid (TFA) content extracted from the two *CrFAP89*-silenced algal cells lines was significantly decreased, by 12.4 and 13.3% compared with the average content of TFA in the control algae. Only four kinds of fatty acids showed an obvious shift in the *CrFAP89*-silenced lines, including C16:0, C16:1, C16:4, and C18:3n3. Among them, only the levels of 9-hexadecenoic acid (C16:1) showed a significant increase in *CrFAP89*-silenced algae between 238.5 and 318.5% compared to the control lines. The most significantly diminished fatty acid was hexadeca-4,7,10,13-tetraenoic acid (C16:4), whose content in *CrFAP89*i lines was decreased between 25.5 and 45.8%. Palmitic acid (C16:0) is the only saturated fatty acid that showed a significant decline in *CrFAP89*i lines, with a percentage decreased ranging from 16.8 to 18.8%.

**Table 1:** Identification and quantification of fatty acids from *CrFAP89*i and control strains after growth on HSM for 9 days.

Fatty acids	*Maa7*-34	*Maa7*-66	*CrFAP89*i-6	*CrFAP89*i-61
C12:0	0.055 ± 0.009	0.067 ± 0.006	0.058 ± 0.002	0.067 ± 0.007
C14:0	0.077 ± 0.009	0.083 ± 0.009	0.078 ± 0.008	0.073 ± 0.007
C16:0	25.287 ± 0.634^a^	25.912 ± 0.578^a^	21.031 ± 0.772^b,∗∗^	21.069 ± 0.329^b,∗∗^
C16:1	2.721 ± 0.076^a^	3.572 ± 0.338^a^	8.518 ± 0.544^b,∗∗^	8.667 ± 0.172^b,∗∗^
C16:2	3.432 ± 0.341	2.541 ± 0.385	2.139 ± 0.103	2.553 ± 0.377
C16:4	14.977 ± 0.843^a^	11.358 ± 0.666^b^	8.462 ± 0.529^c^	8.122 ± 0.598^c^
C18:0	2.092 ± 0.227	2.928 ± 0.102	2.379 ± 0.272	2.545 ± 0.229
C18:1t	12.034 ± 0.613	13.182 ± 0.577	11.584 ± 0.339	11.582 ± 0.392
C18:2t	13.075 ± 0.438	12.374 ± 0.621	11.04 ± 0.645	11.006 ± 0.705
C18:3(5,9,12)	7.941 ± 0.844^a^	15.398 ± 0.751^b^	11.059 ± 0.191^ac^	11.529 ± 0.32^c^
C18:3n3	19.946 ± 0.547^a^	18.979 ± 0.626^a^	14.968 ± 0.223^c^	13.049 ± 0.499^d^
C20:1	0.999 ± 0.129^a^	0.673 ± 0.049^a^	0.48 ± 0.044^b^	0.627 ± 0.103^ab^
TFA	102.637 ± 2.264^a^	107.067 ± 3.664^a^	91.799 ± 2.56^b^	90.888 ± 1.682^b^

*Data shows the average of the strains from three repeated tests, in the form of mean ± standard error of representation. Lines with an empty vector represent a control and TFA represents the total fatty acid content. All data are expressed in mg/g (dry weight). The data were analyzed by SPSS11.5 with one-way analysis of variance (ANOVA), followed by Duncan’s post-test. The different subscript letters indicates that they were significantly different at *P* ≤ 0.05, ^∗∗^*P* ≤ 0.01 according to one-way ANOVA.*

## Discussion

WD40-repeat-containing proteins are found in a wide variety of eukaryotes. Plenty of these proteins have been implicated as regulators in developmental progress and environment sensing (Chuang et al., 2015). With the help of high-throughput sequencing and bioinformatics analysis, 98 WDR proteins from *C. reinhardtii* have been recorded in the UUCD database. Among them, six WDR domain-containing proteins are known to localize in the flagella, namely CrFAP89, CrFAP44, CrFAP187, CrFAP196, CrPF20, and CrODA9 (Gao et al., 2012). A seven-bladed β-propeller fold formed by repeats of WD40 motif is the main feature of these proteins. WDR motifs are considered the crystal interaction sites in many WDR proteins (Ghislain et al., 1996; Pashkova et al., 2010). CrFAP89 encodes for a 1481-amino acid protein that contains fourteen WD40 repeats that form two individual β-sheet structures (**Figure 1D**). These two WDxR maybe the docking sites for E3s. The phylogenetic tree shows that the CrFAP89 from *C. reinhardtii* is homologous to WDR proteins from other eukaryotic species, including NOTCHLESS and Ribosome assembly protein 4 (**Figure 1C**), which plays a role in organ number and size, stomatal index, flowering, and seed development in higher plants and animals (Chantha et al., 2006; Stirnimann et al., 2010; Beckcormier et al., 2014). Thus, we suggest that *C. reinhardtii* FAP89 protein can form two β-propeller structures and most likely has a similar function in regulating the division and development of algal cells.

*Chlamydomonas reinhardtii*, a green biflagellate alga, has been used as a model to study the lipid metabolism, photosynthesis, and ciliary/flagellar function in recent decades. Its flagella have many important functions, such as controlling the motility, the regulation of cell cycle, and environment sensing (Bisova and Zachleder, 2014; Yanagisawa et al., 2014). About 360 flagellar proteins were identified in *C. reinhardtii* through proteomic analysis, including signal transduction proteins, motor proteins, nucleotide metabolism, glycolytic enzymes, malate dehydrogenase, conserved uncharacterized proteins, and predicted membrane proteins (Pazour et al., 2005). Recent studies have demonstrated that ubiquitination is involved in the regulation of flagellar disassembly that occurs prior to the mitosis phase of the cell cycle (Long et al., 2015). Until now, the function of WDR proteins in the flagella of *C. reinhardtii* has been poorly understood. Here, our analysis of CrFAP89 provides a new insight into the function of flagellar proteins. Previous studies have shown there is a striking temporal and functional separation between cell growth and rapid cell division, which is associated with flagellar disassembly (Cross and Umen, 2015). The suppression of *CrFAP89* resulted in an increase in cell number and cell size (**Figure 3**), suggesting that CrFAP89 is essential for the regulation of cell division in *C. reinhardtii*.

Our results also show that *CrFAP89* may be involved in environment sensing in *C. reinhardtii*. Expression of *CrFAP89* significantly increased under nutrient-deficient conditions, especially in the absence of sulfur (**Figure 2**). Nutrition is an important factor affecting the physiology and metabolism of microalgae. Previous studies have shown that *C. reinhardtii* can accumulate 10-fold higher lipid content when deprived of nutrients, with a 90% decrease in biomass (Deng et al., 2011). High-throughput RNA sequencing showed that nitrogen deprivation activated a subset of genes involved in gametogenesis, fatty acid biosynthesis, while down-regulating photosynthesis, protein biosynthesis, glyoxylate cycle, and gluconeogenesis (Miller et al., 2010). Sulfur deprivation has similar effects on *C. reinhardtii*, including lipid accumulation, diminished cell growth, and photosynthesis. Also, it is more suitable for lipid production than nitrogen deprivation, owing to the higher biomass and chlorophyll and TAG content observed in S-deprived samples of *C. reinhardtii* mt(+) and mt(-) strains (Cakmak et al., 2012). Iron is another essential element for cell development. The deprivation of iron usually causes damage to the photosystems, and diminishes photosynthetic capacity (Terauchi et al., 2010). The expression of *CrFAP89* drastically increased under iron deficiency after 2 days (**Figure 2**). Moreover, silencing of CrFAP89 also increased the tolerance of the algal cells to various nutrient-deficient conditions (**Figure 3**). Collectively, these results indicate that CrFAP89 functions as an environment response factor or regulator of signal transduction; however, its mechanism of action is still unknown.

Lipid biosynthesis in algae involves a complex metabolic network pathway. Nitrogen or sulfur deprivation is an effective method to induce algae lipids biosynthesis. The fatty acids composing TAG are mainly long chain fatty acids. C16, C18, and C20 are long chains fatty acids that are accumulated both under nitrogen and sulfur deprivation (Yang et al., 2015). Silencing of the *CrFAP89* gene caused a significant decreased in C16, C18, and C20 fatty acids (**Table 1**), indicating that *CrFAP89* is essential for long chain fatty acid biosynthesis. Previous studies on the *C. reinhardtii* lipid metabolism have revealed that DGAT and PDAT are key enzymes in TAG biosynthesis (Kangsup et al., 2012; Hung et al., 2013), and also have shown that the one type-1 *DGAT*s and five type-2 *DGAT*s are expressed under nitrogen starvation. Except for *DGTT2*, which was constitutively expressed, the other *DGAT* and *PDAT* genes were up-regulated in response to nitrogen starvation (Miller et al., 2010; Msanne et al., 2012). However, a heterologous complementation assay in yeast has shown that among the CrDGATs, DGTT2 is most likely the main isozyme contributing to TAG biosynthesis (Hung et al., 2013). In our study, except for *CrDGTT4*, which was slightly up-regulated in *CrFAP89 RNAi* lines, all other *DGTT* and *PDAT* genes were down-regulated (**Figure 4D**). CrFAP89 regulates lipid synthesis through these DGATs or PDATs via direct or indirect pathways.

## Conclusion

Our results suggest that CrFAP89 functions as regulatory factor for cell growth and lipid accumulation in *C. reinhardtii*. Although the lipid content in the *CrFAP89*-RNAi strain declined, the biomass significantly increased. Furthermore, suppression of *CrFAP89* expression resulted in resistance to nutrition starvation conditions. *CrFAP89*-RNAi strains maybe suitable for multiple lipid synthesis by gene transformation, and to obtain ideal strains that grow better and accumulate more lipids. Further research is necessary to elucidate the complexes that interact with CrFAP89, which may function in regulating diverse cellular processes.

## Author Contributions

QL and ZH conceived and designed the experiments. QL, WS, YL, and CW performed the experiments and contributed to the interpretation of the results. QL analyzed the data and wrote the manuscript. CW and ZL revised the manuscript. All authors have read and approved the final manuscript.

## Conflict of Interest Statement

The authors declare that the research was conducted in the absence of any commercial or financial relationships that could be construed as a potential conflict of interest.

## Footnotes

1 https://phytozome.jgi.doe.gov/pz/portal.html

2 http://smart.embl-heidelberg.de/

3 http://www.uniprot.org/

4 http://www.expasy.org/

5 http://www.sbg.bio.ic.ac.uk/phyre2/html
