# Supplementary material for: Flagella-Associated WDR-Containing Protein CrFAP89 Regulates Growth and Lipid Accumulation in Chlamydomonas reinhardtii
Source: Front Plant Sci. 2018 May 29;9:691. doi: 10.3389/fpls.2018.00691 (PMC5987165; doi:10.3389/fpls.2018.00691)
Supplement: Supplementary file 1 [file Table_1.docx]

**Supplemental Table S1. Components of stressful mediums.**

| Mediums  Components (mg/L) | **HSM** | **HSM-N** | **HSM-S** | **HSM-Fe** |
| --- | --- | --- | --- | --- |
| Sodium acetate (hydrate) | 2,000 | 2,000 | 2,000 | 2,000 |
| NH_4_Cl | 500 | - | 500 | 500 |
| NaCl | - | 546.7 | - | - |
| MgSO_4_·7H_2_O | 20 | 20 | - | 20 |
| MgCl_2_.6H_2_O | - | - | 16.5 | - |
| CaCl_2_·2H_2_O | 10 | 10 | 10 | 10 |
| K_2_HPO_4_ | 1,440 | 1,440 | 1,440 | 1,440 |
| KH_2_PO_4_ | 720 | 720 | 720 | 720 |
| KCl | - | - | - | - |
| NaH_2_PO_4_ | - | - | - | - |
| Na_2_HPO_4_ | - | - | - | - |
| EDTA-Na_2_ | 50.0 | 50.0 | 50.0 | 50.0 |
| ZnSO4·7 H2O | 22.0 | 22.0 | 22.0 | 22.0 |
| H_3_BO_3_ | 11.4 | 11.4 | 11.4 | 11.4 |
| MnCl_2_·4 H2O | 5.1 | 5.1 | 5.1 | 5.1 |
| FeSO_4_·7 H2O | 5.0 | 5.0 | 5.0 | - |
| CoCl2·6 H2O | 1.6 | 1.6 | 1.6 | 1.6 |
| CuSO4•5 H2O | 1.6 | 1.6 | 1.6 | 1.6 |
| (NH_4_)_6_ MO_7_O_24_·4 H2O | 1.1 | 1.1 | 1.1 | 1.1 |
| Glucose | - | - | - | - |
| Sorbital | - | - | - | - |
