# Supplementary material for: Flagella-Associated WDR-Containing Protein CrFAP89 Regulates Growth and Lipid Accumulation in Chlamydomonas reinhardtii
Source: Front Plant Sci. 2018 May 29;9:691. doi: 10.3389/fpls.2018.00691 (PMC5987165; doi:10.3389/fpls.2018.00691)
Supplement: Supplementary file 2 [file Table_2.DOCX]

**Supplemental Table S2. Primers used for qPCR assays.**

| **Gene name** | **Primer sequences** |
| --- | --- |
| CrFAP89 | F: 5’-AAGACACTGGCGCTGGTGGACG |
| CrFAP89 | R: 5’-GCGATTGCAGCCGCAGACTTGG |
| CrDGAT1 | F: 5’-ACTGGTGGAATGCGGCTAC-3’ |
| CrDGAT1 | R: 5’-TAGCAGCTCGTGGAACACAG-3’ |
| CrDGTT1 | F: 5’-GAAGCAGGTGTTTGGCTTCT-3’ |
| CrDGTT1 | R: 5’-CAGTGCCTCCGTGTAGGTCT-3’ |
| CrDGTT2 | F: 5’-GCGCCGCAACATTTACATGG-3’ |
| CrDGTT2 | R: 5’-CAGCCGTACTCGGTCTTGTG-3’ |
| CrDGTT3 | F: 5’-GTCAGAGCCAAGTGCTGGAC-3’ |
| CrDGTT3 | R: 5’-TCCACCTCCTTGTCGAACTC-3’ |
| CrDGTT4 | F: 5’-GCATGTTTGGGCAGTACGGC-3’ |
| CrDGTT4 | R: 5’-GCCTTGTGCTTGTCGTACAG-3’ |
| CrDGTT5 | F: 5’-AGTCACTGCAGCAGCTGTCG-3’ |
| CrDGTT5 | R: 5’-GCCCACACACATCATGAGCG-3’ |
| CrPEPC1 | F: 5’-AGCTGGGCCGCCTCAACAT-3’ |
| CrPEPC1 | R: 5’-TTGTCGATGGCCGCCTCCA-3’ |
| 18S rRNA | F: 5’-TCAACTTTCGATGGTAGGATAGTG-3’ |
| 18S rRNA | R: 5’-CCGTGTCAGGATTGGGTAATTT-3’ |
